# Early Life Experiences and Exercise Associate with Canine Anxieties

**DOI:** 10.1371/journal.pone.0141907

**Published:** 2015-11-03

**Authors:** Katriina Tiira, Hannes Lohi

**Affiliations:** 1 Department of Veterinary Biosciences and Research Programs Unit, Molecular Neurology, P.O.Box 63, 00014 University of Helsinki, Helsinki, Finland; 2 The Folkhälsan Research Center, Helsinki, Finland; Technion - Israel Institute of Technology, ISRAEL

## Abstract

Personality and anxiety disorders across species are affected by genetic and environmental factors. Shyness-boldness personality continuum exists across species, including the domestic dog, with a large within- and across-breed variation. Domestic dogs are also diagnosed for several anxiety-related behavioral conditions, such as generalized anxiety disorders, phobias, and separation anxiety. Genetic and environmental factors contributing to personality and anxiety are largely unknown. We collected questionnaire data from a Finnish family dog population (N = 3264) in order to study the associating environmental factors for canine fearfulness, noise sensitivity, and separation anxiety. Early life experiences and exercise were found to associate with anxiety prevalence. We found that fearful dogs had less socialization experiences (p = 0.002) and lower quality of maternal care (p < 0.0001) during puppyhood. Surprisingly, the largest environmental factor associating with noise sensitivity (p < 0.0001) and separation anxiety (p = 0.007) was the amount of daily exercise; dogs with noise sensitivity and separation anxiety had less daily exercise. Our findings suggest that dogs share many of the same environmental factors that contribute to anxiety in other species as well, such as humans and rodents. Our study highlights the importance of early life experiences, especially the quality of maternal care and daily exercise for the welfare and management of the dogs, and reveals important confounding factors to be considered in the genetic characterization of canine anxiety.

## Introduction

Large and stable personality differences (also called coping styles, temperament, behavioral syndrome) are observed in many behavioral traits, such as in aggressiveness or fearfulness across species [1–2]. However, although the personality variation is well-documented in many species, the ontogeny and development of personality is less studied [3]. Personality dimensions have high heritability estimates (h^2^ = 0.3–0.5) [4–6] however, environmental factors also have a large contribution. Parallel to the study of genetics of personality, we also need information on the environmental factors that might affect the development of various personalities. In this study, we will investigate environmental factors that associate with fearfulness in privately owned family dogs.

A dog is included in every third household in Finland, and the estimated worldwide population size of dogs varies from 700,000,000 to one billion [7]. Canine personality has a large impact on both the canine’s and the owner’s welfare. Aggressiveness is often motivated by fear, and bite injuries resulting from human-directed aggression could be considered an important public health concern. Domestic dogs are also diagnosed for several anxiety-related behavioral conditions, such as generalized anxiety disorders, phobias, and separation anxiety, which in some cases can be considered as severe welfare issues in dogs [8]. Fearful dogs are also not suitable to be trained as working dogs [9].

Fear and anxiety are both emotions with negative valence [10]. Fear is suggested to be brief in duration, stimulated by a specific stimuli, and resulting in either fight or flight, whereas anxiety is prolonged, focused on the future, and does not necessarily have a specific object of threat [11–13]. In dogs, fearfulness can be categorized based on the object and the situation into social and non-social fearfulness [14]. The social category includes fear of unfamiliar people and dogs, whereas the non-social fear –category includes fear of different objects such as new situations, loud noises (noise phobia / noise sensitivity), heights, or shiny/slippery floors. In the literature, fear of loud noises is often referred to as noise phobia because of extreme panic reactions in some cases. However, we prefer to use the proposed term ‘noise sensitivity’ [15], since often fearful behavioral reactions towards loud noises, such as thunder storms, fireworks or gun shots, do not fulfill the criteria of phobia. Separation anxiety in dogs refers to a behavior that includes signs of anxiety, fear, or phobia expressed by a dog when separated from the owner [15].

Fearfulness and noise sensitivity have relatively high heritability [16–18], but are largely affected also by the environment. Two major environmental factors known to affect general fearfulness in dogs include lack of juvenile experiences and aversive learning. Deficits in early socialization [19–21] and unpleasant experiences [22] at any age affect a dog’s fearfulness. Noise sensitivity is often thought to occur as a consequence of adverse experiences, however, other mechanisms are also most likely involved in the development of this problem [23,24]. Only a few environmental factors, such as being the owner's first dog [25], being a sterilized female [26], or having shorter daily walks and fewer activities [27] have been found to correlate with noise sensitivity. Interestingly, the effects of the quality of maternal care on fearfulness or noise sensitivity has not been investigated in dogs earlier, despite its importance on developing personality in other species [28,29] and the fact that a family dog’s breeding system allows detailed observation on the maternal care.

Dog is suggested to be a natural animal model for many complex human traits, behavior included, [30,31] due to the unique population history and genetic architecture of the breeds. The overall aim of our research is to find loci responsible for various anxiety traits in dogs and towards this aim, we have earlier developed and validated an owner-filled questionnaire survey designed for behavioral genetic sample collection [32]. The main aim of this study was to investigate associated environmental factors in fear-related behaviors in family dogs using a validated owner-filled questionnaire. This study reveals associated environmental factors that are useful not only for genetic studies, but are also important factors to be considered for canine welfare.

## Methods

### Data collection

Data on dog behavior and environmental factors was collected using a validated owner-filled questionnaire, (S1 Appendix) which has been earlier shown to correlate with dogs' behavior in test situations (external validity) and to have good test-retest reliability [32]. The questionnaire includes 35 questions (S1 Appendix) altogether. The potential fearful reaction and frequency (0–4) towards strangers, unfamiliar dogs, and new situations were asked (S1 Appendix) [32]. To reduce the possible subjectivity of the owner’s judgment, we added a question for the owners to describe ‘*how exactly’* the dog behaved in a specific situation. If the owner felt that the dog was fearful when meeting a stranger (or strange dogs, and/or novel situations), the owner had to indicate a specific reaction: *how* did the dog behave (for example, the dog withdraws when meeting a stranger). Similarly, if the owner felt that the dog did not show fear towards a stranger, a more specific description of the reactions was required to enable our own evaluation of the situation. During data collection, the questionnaire was modified three times, resulting in four slightly different versions of the questionnaire (the first one being a paper version, the three others—online questionnaires). The main questions regarding our target traits, fearfulness (towards persons, dogs, and new situations), noise sensitivity, and separation anxiety have not changed between the versions. The main difference comes from adding further background questions to versions three and four (maternal care, place of birth, type of food, extra nutrients, time spent alone/day, daily exercise) to better document the early life experiences and conditions of the dogs. We have also removed one unclear question that continuously confused owners (question on fearfulness towards novel objects), which is also not included in the following analysis. The latest version also has more detailed questions on ‘bold’ behavior. Several background questions regarding early life experiences, such as socialization and the puppy period, were included, as well as more general questions concerning daily routines and diet, with food types and extra nutrients. Instead of trying to capture the entire spectrum of phenotypic variation in fearfulness, we aimed to structure the questionnaire so that it would find the most fearful individuals as 'cases', and those with no marked fear reactions as 'controls'. We derived several behavioral variables from the questionnaire data (Table 1), which were used as response variables in analyzing the associated environmental factors.

**Table 1:** Behavioral and environmental variables derived from questionnaire data.

Variable	Explanation
Fear-status	Binomial (case/control) variable, where the case dog shows fear either towards strangers or in new situations/places, or unfamiliar dogs at minimum 40% of situations and at maximum 100% in both situations. Control dogs are not reported to show fear in any of the above-mentioned circumstances or fear towards strange dogs. The decision to group these social and situation anxieties is based on the initial inspection of data, where all these fears were observed to be significantly correlated (Human & New situation r_s_ = 0.423, p<0.0001 N = 3068; Humans & Dogs r_s_ = 0.420, p<0.0001 N = 3038; Dogs & New situations r_s_ = 0.332, p<0.0001 N = 3009).
Noise sensitivity –status	Binomial (case/control) variable, where case dogs have noise reactivity >0 towards loud noises (thunder, fireworks, gunshot). Control dogs are not reported to show fear in any of the above-mentioned circumstances.
Noise reactivity score	Categorical variable. Describes the frequency and intensity of fearful reactions towards loud noise. Calculated as follows: (sum of fearful behavioral reactions to fireworks) x frequency of fear reaction to fireworks + (sum of fearful behavioral reactions to thunder) x frequency of fear reaction to thunder + (sum of fearful behavioral reactions to gunshot) x frequency of fear reaction to gunshot.
Shyness score	Categorical variable. Describes the frequency showing fear towards unfamiliar people, dogs and in new situations. Calculated as sum of frequencies of showing fear towards strangers + dogs + in new situations.
Separation anxiety -status	Binomial (case/control) variable, which is based only on one question “Does your dog show separation anxiety?” Answers stating that the owners are not certain were excluded from the analysis.
Socialization (Environmental factor)	Categorical variable. The sum variable of frequencies of how much the dog met strange women, men, children, dogs, visited the city, travelled by car or bus, when the dog was between 8–12 weeks of age.
Maternal care quality (Environmental factor)	Categorical variable, which is based on the answers on the question "How did the mother of your dog took care of the puppies?", the owner could choose from the following statements, which were graded into 1–7 scale: (*Mother took extremely good care of the puppies and spent a lot of time with the puppies / Mother took good care of the puppies / Mother took relatively good care of the puppies*, *but sometimes it had to be pushed back to spend time with the puppies / At the beginning*, *the mother spent some time with its puppies*, *but later started to avoid being with the puppies / Mother did not want to spent time with the puppies*, *even at the beginning*, *but nursed enough*, *so the puppies were not taken from its mother / Mother did not take care of puppies; puppies were taken to a surrogate mother*, *or they were bottle fed*). In addition, the owner could choose *"I don’t know*," or *"If any of the options was not suitable*, *please tell in your own words*.*"* If the owners only described the behavior in their own words, it was translated into a categorical number (1–7) based on the evaluation of KT.
Exercise (Environmental factor)	Categorical variable. The daily amount of exercise was calculated including the daily time reported in exercise and multiplying this with either: 1 (dog mostly in leash), 2 (part of the walk free and part of the walk in leash), or 4 (free during most of the walk).

We advertised the questionnaire to owners of all breeds, but especially Great Danes, German Shepherds, Belgium Shepherds, Staffordshire Bull terriers, Lagotto Romagnolos and Salukis. This is because we aimed for large sample sizes from breeds with a large number of existing blood samples already in our Dog DNA bank. The questionnaire was advertised via breed clubs and Facebook. Both shy and bold individuals, as well as dogs with fearful reactions to loud noises, and also dogs with no marked behavioral reactions to loud noises were invited to participate.

### Statistical analysis and used behavioral variables

We analyzed the possible association of environmental factors with several anxieties using the generalized linear mixed model (GLMMs) with binomial distribution (PROC GLIMMIX, SAS version 9.3), using breed and the questionnaire-version (1–4) as a random variable (all breeds were pooled). The GLIMMIX procedure allows the analysis of fixed and random variables in the same model, and it also allows non-normal data and correlation among the variables. The response variable for fearfulness was Fear-status, for noise sensitivity—Noise sensitivity –status and for separation anxiety we used Separation anxiety –status (referred hereafter as A analysis) (Table 1). Fear –status was determined such that as case dogs we had individuals experiencing fear more than in 40% of situations met (unfamiliar people and situations); correspondingly, control dogs did not show fear towards unfamiliar people, dogs or situations (Table 1). All dogs having Noise reactivity > 0 were assigned to Noise sensitivity cases (Table 1). That means that any dogs showing any fearful reaction, even mild, towards thunder, fireworks and gunshots were considered to belong to the noise sensitivity case group.

Often the dog, which is generally fearful, also has noise sensitivity, and/or separation anxiety. Thus, in the A analysis, a case dog for one anxiety may also have other anxieties as well. To investigate those environmental factors that contribute to one specific anxiety-type, we re-analyzed the data. In these B analyses, we included dogs having *only one specific* anxiety-type (either fearfulness, noise sensitivity, or separation anxiety) as cases, and controls were dogs that had no anxieties (dogs with Fear-status = 0, Noise sensitivity = 0 and Separation anxiety = 0). Finally, we compared whether environmental factors would differ between those dogs that have *all* three comorbid anxieties (dogs have Fear-status = 1, Noise sensitivity = 0 and Separation anxiety = 1) and between dogs having no anxieties (dogs have Fear-status = 0, Noise sensitivity = 0 and Separation anxiety = 0). This analysis is referred to as C analysis.

In all analyses, the following explanatory variables were included as fixed variables in the analysis: *sex*, *size of the dog*, *age of arrival to a new home (in weeks)*, *socialization* (amount of socialization after the puppy has arrived into the new home, in Finland the average age for this is 7–8 weeks, see Table 1), *number of children in the household*, *number of adults*, *number of dogs in the household*, *neutering status*, *kennel/indoor dog*, *was the dog the owner’s first*, *second*, *etc*, *the time the dog has to spend alone during a normal day*, *amount of daily exercise* (see Table 1), *amount of activities done with the dog*, *dietary supplements* (does the dog get any dietary supplements yes/no), *type of food (industrial/home made)*, *quality of maternal care*, *age of separation from the mother*, *problems at birth* (yes/no). Non-significant terms were dropped out individually and only the statistically significant terms were kept. The size of the dog was estimated for each dog as the average breed-specific height of that particular sex of particular breed. Therefore, no height was estimated for mixed breeds dogs. Multicolinearity was assessed in all our final generalized linear mixed models using PROC REG analysis. Factor *place of birth (at the dog’s permanent home/ breeder’s place)* was not included into the generalized linear mixed model, as although included in all versions of the questionnaire, it included many missing values or “don’t know” answers and therefore including this variable into the model would have reduced the sample size significantly. We therefore analyzed *the place of birth*–variable separately using Wilcoxon rank sum test, comparing the Shyness and Noise reactivity -scores (see Table 1) between dogs that were born at their permanent home or at the breeder’s place. Kruskall-Wallis test and Spearman correlation were used to investigate the association between daily exercise and other variables.

### Ethics statement

The data in this study is collected using an online questionnaire. Before completing the questionnaire, owners were informed of the following: “*all filled questionnaires are included in the study*, *which focuses on the phenotypic characteristics of fear and noise phobia*, *and possible associated environmental factors*. *All information submitted is kept strictly confidential*. *Neither you nor your dog(s) will be identified during the study*”. By answering the questionnaire, owners thus gave permission to use the data in studying the genetic and environmental factors affecting anxiety-related traits."

## Results

### Demographics

We received altogether 3284 answers (questionnaire versions 1 & 2–1405 and versions 3 & 4–1878 answers) from 192 breeds. Six breeds had over 100 replies (Border Collie, Lagotto Romagnolo, German Shepherd, Saluki, and Great Dane, Belgian Shepherd Tervueren & Groenendal), in addition, three breeds had 80–100 replies (Bearded Collie, Staffordshire Bull Terrier, and Shetland Sheepdog). The age of the dogs varied from 3 months to 15 years, where the mean age was 5.2 years ± 3.3, (median 5 years); however in the following analysis, we included only dogs older than 6 months. This left us with a total of 3262 dogs, from which there were only 19 dogs (6–12 months) that were under 1 year old. Data consisted of 1737 females (mean age 5.3 years ± 3.3, N = 1717) and 1525 males (mean age 5.2 years ± 3.2, N = 1507).

### Environmental factors associated with fearfulness

Potential differences in environmental factors between case and control dogs were analyzed in two different ways (A & B analysis) to account for possible anxiety comorbidity. We found several environmental factors to differ between fearful and non-fearful dogs (A analysis, Table 2a). In the generalized linear model analysis, maternal care and the amount of socialization had the largest effects, indicating that fearful dogs had received poorer maternal care and were less socialized compared with non-fearful dogs. Fearful dogs also lived in households with less conspecifics and with more adults, were younger, and more often females compared with control dogs (Table 2a). We also found that dogs that lived indoors had higher scores for fearfulness compared with dogs either living both indoors and outside, and dogs living mainly outside. The amount of daily exercise was not statistically significant in the model, however, there was a tendency that fearful dogs get less exercise compared with non-fearful dogs. Dogs' birthplace was analysed separately, and the results suggest that dogs born (in non-permanent homes) at the breeder’s places (N = 384) were less fearful compared with dogs born at their permanent homes (N = 1238) (Z = -2.26, p = 0.024). We further analysed whether the quality of maternal care differed between these two birthplaces, and found that mothers that gave birth in their permanent homes (N = 920) took better care of their offspring, compared with mothers that were taken to breeders' homes (N = 291) to give birth (Z = -2.25, p = 0.025).

**Table 2:** Generalized linear mixed model analysis results on the association between environmental factors and fearfulness.

**a) Fear-status (A)**	**Num df**	**Den df**	**F Value**	**P value** ^**1**^
*Age (in years)*	1	780	8.91	**0.0029**
*Number of dogs in the family*	1	780	7.50	**0.0063**
*Socialization*	1	780	14.29	**0.0002**
*Maternal care*	1	780	19.43	**<0.0001**
*Sex*	1	780	7.45	**0.0065**
*Number of adults in the family*	1	780	5.93	**0.0151**
*Indoors/kennel/both*	2	780	3.74	**0.0242**
*Daily exercise*	1	780	3.57	0.0593
**b) Fear-status (B)**	**Num df**	**Den df**	**F Value**	**P value** ^**1**^
*Age (in years)*	1	591	4.92	**0.0269**
*Socialization*	1	591	9.61	**0.0020**
*Activities*	1	591	9.59	**0.0020**
*Daily Exercise*	1	591	8.09	**0.0020**
*Age of separation from the mother*	1	591	2.91	0.0884

a) in A analysis, the variable Fear-status is used as a binary response variable. Fit statistics: Gener. Chi-Square / df = 0.96

b) In B analysis, the variable Fear-status is used as a binary response variable, however, we have excluded all dogs that show a fearful reaction towards loud noises, and also dogs reported to have separation anxiety. Fit statistics: Gener. Chi-Square / df = 0.85

Both the breed and the questionnaire version were used as a random factor in both analyses, and variables were reduced from the model one by one.

Results of the type III analysis are presented.

^1^Only variables with statistical significance <0.1 are presented, variables with significance <0.05 are in bold.

The results of the B analysis controlling comorbidity identified three environmental factors and suggest that *solely* fearful dogs are younger, less socialized, and get less daily exercise compared to non-fearful dogs. Owners also engaged less in various activities with fearful dogs (Table 2b).

### Environmental factors associated with noise sensitivity

The largest explanatory factor associating with noise sensitivity in the A analysis was daily exercise. Dogs with noise sensitivity got significantly less daily exercise compared with dogs with no noise sensitivity (Table 3a). In addition, sterilized dogs, and owners’ first dogs had more noise sensitivity. Although the sex was not a significant factor in the model, interaction between sex and sterilization status was—intact females were sensitive more often compared with intact males (only intact dogs included, noise sensitivity status * sex, Chi-Square = 4.85, p = 0.03; only sterilized individuals included, noise sensitivity status * sex, Chi-Square = 0.30, ns.) We also found that dogs with noise sensitivity were older and spent slightly less time alone on an average day compared to controls (Table 3a).

**Table 3:** Generalized linear mixed model analysis results on the association between environmental factors and noise sensitivity.

**a) Noise sensitivity (A)**	**Num df**	**Den df**	**F Value**	**P value** ^**1**^
*Age (in years)*	1	1513	11.98	**0.0006**
*Owners 1st*, *2nd*, *etc*. *dog*	1	1513	10.23	**0.0014**
*Daily exercise*	1	1513	22.07	**<0.0001**
*Sterilized*	1	1513	10.10	**0.0015**
Sex * Sterilized	1	1513	5.56	**0.0039**
*Time spent alone/day*	1	1513	4.86	**0.0276**
**b) Noise sensitivity (B)**	**Num df**	**Den df**	**F Value**	**P value** ^**1**^
*Age (in years)*	1	657	16.24	**<0.0001**
*Daily exercise*	1	657	13.96	**0.0002**
*Time spent alone/day*	1	657	9.66	**0.0020**
*Socialization*	1	657	3.95	**0.0472**
*Number of dogs in the family*	1	657	3.62	0.0576

a) In A analysis, the variable Noise sensitivity-status is used as a binary response variable. Fit statistics: Gener. Chi-Square / df = 0.98

b) In B analysis, the variable Noise sensitivity-status is used as a binary response variable; however, we have excluded all dogs that that show a fearful reaction either towards strangers or in new situations/places at a minimum of 40% of occasions and also dogs reported to have separation anxiety. Fit statistics: Gener. Chi-Square / df = 0.94

Both the breed and the questionnaire version were used as a random factor in both analyses, and variables were reduced from the model one by one.

Results of the type III analysis are presented.

^1^Only variables with statistical significance <0.1 are presented, variables with significance <0.05 are in bold.

We further analysed the role of daily exercise against other factors in a dog’s life. We found that daily exercise did not correlate with puppy socialization (r_s_ = 0.01, p = ns, N = 1635), or aggressiveness towards strangers (r_s_ = -0.03, p = ns, N = 1725), but it had a modest negative correlation with aggressiveness towards dogs (r_s_ = -0.05, p = 0.03, N = 1725) and towards the owner (r_s_ = -0.09, p = 0.002, N = 1725). Also, the more the dog had daily exercise, the less time it spent alone (r_s_ = -0.07, p = 0.003, N = 1722), the more the owner spent time on activities with the dog (r_s_ = 0.21, p < 0.0001, N = 1689), and also dogs having a lot of daily exercise had arrived at an earlier age to their current homes (r_s_ = -0.07, p = 0.005, N = 1722). There were also slightly more children (r_s_ = 0.06, p = 0.02, N = 1708) in families that exercised their dogs more. The daily exercise variable was formed as multiplication of daily time spent (min) and the exercise type (dog on the leash all the time (1), partly (2), or free all the time (4)) (Table 1). Since the exercise type (free/on leash) has a large effect on the exercise –variable, we wanted to further investigate whether environmental factors differ between dogs that mostly walked free or always on leash. Environmental factors such as age (Z_1,806_ = 0.022, ns), puppy socialization (Z_1,770_ = 0.18, ns), or time spent alone/day ((Z_1,818_ = 1.38, ns) did not differ between these exercise-groups. However, we found that free exercising dogs had more dog companions at home (Z_1,796_ = -10.85, p < 0.0001), and their owners had previously owned more dogs compared with dogs always walking on the leash (Z_1,821_ = -8.59, p < 0.0001). Also, among noise sensitive dogs, the fear of loud noises in free-exercising dogs started later compared with dogs that are always on leash (Z_1, 113_ = -2.68, p = 0.009).

The B analysis, comparing dogs having *only* noise sensitivity and no other anxiety comorbidities, revealed (Table 3b), that noise sensitive dogs got less daily exercise, were older, were less socialized, and spent slightly less time alone on an average day compared to controls (Table 3b). There was also a non-significant tendency that noise-sensitive dogs had less canine companions at home compared to controls. The dogs' birthplace was analysed separately, and the results suggest that dogs born at breeders' places (N = 384) had less noise reactivity compared with dogs born at their permanent homes (N = 1238) (Z = -2.18, p = 0.029).

The onset age of noise sensitivity was reported for 407 dogs. The more dogs the owner had at present (r_s_ = 0.13, p = 0.011, N = 364) and the more they had previously owned (r_s_ = 0.14, p = 0.006, N = 376), the later the fear of loud noises started.

### Environmental factors associated with separation anxiety

Separation anxiety was asked about in only one question in the questionnaire: “Does your dog have separation anxiety?” The A analysis compared the environmental factors between dogs whose owners answered “yes” with dogs whose owners had marked “no”. Dogs with separation anxiety exercised less compared with dogs that did not have separation anxiety (Table 4). *The age of arrival to a new home*–factor had an insignificant tendency to differ between groups (p = 0.052), where dogs with SA had arrived to new homes at an older age. B analysis, comparing dogs having *only* separation anxiety (and no comorbidities), did not find any environmental factors that would be associated with separation anxiety per se.

**Table 4:** Generalized linear mixed model analysis results on the association between environmental factors and separation anxiety.

a) Separation anxiety (A)	Num df	Den df	F Value	P value^1^
*Daily exercise*	1	1551	11.54	**0.0007**
*Age of arrival*	1	1551	3.96	**0.0468**

a) In A analysis, the variable Separation anxiety-status is used as a binary response variable. Fit statistics: Gener. Chi-Square / df = 0.92

Both the breed and the questionnaire version were used as a random factor in both analyses, and variables were reduced from the model one by one.

Results of the type III analysis are presented.

^1^Only variables with statistical significance <0.1 are presented, variables with significance <0.05 are in bold.

### Environmental factors and anxiety comorbidity

We also studied the role of environmental factors for comorbid dogs by comparing dogs that were fearful, had noise sensitivity, and separation anxiety against dogs that were reported not to have any anxieties (C analysis). Dogs having several anxieties had experienced poorer maternal care and were separated later from their mothers; in addition, these dogs spend less time alone and engaged in various activities with the owner less often (Table 5). However, the fit statistics of this analysis (generalized chi-square/DF) was only 0.66 (the closer to 1, the better), which means that results should be interpreted cautiously.

**Table 5:** Generalized linear mixed model analysis results on the association between environmental factors and comorbid anxieties.

a) All anxieties (C)	Num df	Den df	F Value	P value^1^
*Maternal care*	1	431	16.69	**<0.0001**
*Time spent alone/day*	1	431	3.93	**0.0480**
*Activities*	1	431	4.13	**0.0427**
*Age of separation from the mother*	1	431	5.83	**0.0162**

a) Results of the C analysis estimating the association of environmental factors comparing dogs with all anxieties (fearfulness, noise sensitivity, separation anxiety) with dogs with no above-mentioned anxieties reported.

Both the breed and the questionnaire version were used as a random factor in both analyses, and variables were reduced from the model one by one.

Results of the type III analysis are presented.

^1^Only variables with statistical significance <0.1 are presented, variables with significance <0.05 are in bold.

## Discussion

Behavioral traits are affected by a complex interplay of genetic and environmental factors. One of the major benefits of studying family dogs instead of wild animals is the possibility to collect life-long information and estimates on behavior in various situations and environments. Dogs are family members and under constant daily surveillance of the owners, which permits accurate descriptions of many life events. Owners are capable of describing a dog’s behavior, and fearful personality traits in particular, reliably [32–34]. Furthermore, subjective interpretations can be at least partially controlled by the means of presenting the questions in surveys. In this study, we found several early life—and daily routine factors that associate with anxiety and which may have implications on dogs' welfare and strategies for future genetic studies.

### Early life experiences have a major role in the development of a fearful personality

The largest explanatory factors associating with fearfulness were maternal care and the amount of socialization during the puppyhood. Fearful dogs had experienced poorer maternal care and had been less socialized before the first three months of life. When comparing environmental factors between *solely* fearful (without other anxieties) and non-fearful dogs, we did not however find any differences in maternal care, suggesting that maternal care may be especially important in the development of anxiety comorbidities. This was further supported by the observation that maternal care differed in the analysis that compared a combined comorbidity anxiety group (fearfulness, noise sensitivity, and separation anxiety) with non-fearful dogs. We have also found earlier that dogs with stereotypic tail-chasing experienced poorer quality maternal care and had shyer personalities compared with dogs with no tail-chasing [35]. It is good, however, to keep in mind that in this study, the information on the maternal care quality came from the owners and not from the breeders. Some owners had contacted the breeder for further information on the maternal care, and some reported what they had witnessed when visiting the breeder before adopting the puppy. Not all responders were able to answer this question. The question on the quality of maternal care reached good test-retest reliability values when we earlier validated our questionnaire (maternal care quality: Spearman r_s_ = 0.82, p = 0.0002, N = 15) [32]. Even though detailed behavioral observations of maternal care quality together with the later assessment of personality are definitely needed to verify our results, our observation serves as the first indicator of the magnitude of the effect of maternal care quality on the fearfulness in dogs. Early life experiences are well-known to have a large effect on behavior in all animals [36–39] including dogs [36]. Variation in the quality of maternal care has shown to have dramatic changes through epigenetic modifications in stress-coping behavior in rats [28,29], humans, and primates [40,41]. However, the research on maternal care and maternal behavior is completely lacking in dogs. It is well-established that maternal behavior itself has a heritable component [40], and breed-specific differences in maternal care have been observed in other domesticated species [42]. It should not therefore be surprising to find that dog breeds may also differ markedly in maternal behavior, considering the extensive existing variation in morphology and behavior between dog breeds. Domestication and human interference with maternal care may cause relaxed selection on maternal care, and severe defects, and even lack of maternal behavior has been observed in domesticated species [42,43]. Variation in maternal care through epigenetic or genetic factors may greatly contribute to anxiety-related behaviors in dogs.

The fact that puppy socialization was found as a major explanatory factor for fearfulness was not surprising. [19,44]. In Finland, puppies are routinely adopted to new homes at the age of 7–8 weeks, unlike many other countries, where puppies arrive to new homes between 10–12 weeks. The question on the amount of socialization reached very high test-retest reliability values on the validation of our questionnaire (socialization: Spearman r_s_ = 0.94, p<0.0001, N = 34) [32], and it seems that owners are well able to remember the socialization events from the puppy period. Several studies have suggested that early environment and socialization affect avoidance behavior and aggression in dogs [19–21]. Dogs have a sensitive period during approximately 3–12 weeks of age, where life experiences and events have pronounced effects on later behavior [19,21]. The timing and the amount of experiences needed for a balanced personality may, however, vary across breeds.

### Exercise—a major stress resilience factor?

Interestingly, the daily amount of exercise was the largest factor associated with noise sensitivity; dogs with noise sensitivity exercised significantly less compared with dogs with no noise sensitivity. Similarly, daily exercise was the largest environmental factor that was significantly different between dogs having separation anxiety and dogs with no observed symptoms. In addition, daily exercise differed significantly between *solely* fearful and non-fearful dogs (B analysis), and it was also nearly significant between fearful and non-fearful dogs (A analysis). Daily exercise-variable consisted of the minutes per day of walking multiplied by the exercise type (mostly free/ partly on leash/ all the time on leash). If analysed independently, both variables correlate negatively with noise sensitivity. The amount of daily walks was also found earlier to associate with fearfulness; dogs with shorter daily walks had more fear of loud noises, strangers, and startling stimuli compared with dogs with longer walks [27]. Exercise may work as stress resilience, and owners may therefore observe less fearful reactions. This hypothesis is also supported by the observation that dogs that are allowed to exercise freely had later onset noise sensitivity. The resilient effect of exercise on anxiety and depression has been recognized in humans as well [45,46]. The complete underlying mechanism behind this effect of exercise is not clear, but it is known that exercise increases serotonin production both in animals and humans, thus functioning as an antidepressant [47]. Recent studies also showed a reduction in oxidative stress and anxiety-like behavior as a consequence of moderate treadmill exercise in rodents [48]. Our study highlights the importance of exercise in dogs' welfare and warrants further studies, including potential anxiolytic effects through the serotonin pathway.

The alternative explanation for the observed association between exercise and anxiety could relate to the owner factor: anxious dogs are exercised less because fearfulness challenges such routines. These dogs may, for example, run away, be less obedient, or behave aggressively when startled. However, Tami et al. did not find any difference in obedience between dogs with short compared with long walks [27]. Our results did not find any association between daily exercise and aggressiveness towards strangers, but we did find a modest association where dogs with less daily exercise were more aggressive to other dogs. We could speculate that these dogs may also have fewer experiences (due to shorter walks) and therefore be more fearful. However, puppy socialization did not correlate with daily walks. Thus, our and others' data makes the alternative explanation for the observed association less likely. However, daily exercise was also positively associated with other routines that together may have an effect on anxiety. Dogs that got more daily exercise spend less time alone, and their owners had more activities with these dogs (training, etc) as well. The amount of daily exercise can thus be a variable that indicates the overall quality of general dog management.

A recent study suggested an association between dogs' morphology and several behavioral characteristics [49]. They found a strong negative correlation between body-size and unwanted behaviors—smaller dogs had significantly more compulsive behaviors, mounting behavior, separation-related problems, urine marking, but most importantly, dog-directed fear, and non-social fear [49]. Our results do not support their finding. We included the size of the dog (average height of that breed) into all analyses, however, it was significant only when daily exercise was excluded from the analysis. Further analysis showed that smaller dogs are more often kept on leash compared with larger dogs (χ2_2,1720_ = 108,03, p < 0.0001), and also get less exercise (r_s_ = 0.30, p < 0.0001, N = 1708). Thus, the *size* of the dog may not be a major factor for fearfulness, but instead, small dogs most likely have different daily routines, such as exercise, which may confound results, if variables such as exercise are excluded.

### Age, other dogs, and the effect of the owner

Fearful dogs were observed to be younger compared with non-fearful dogs; however, dogs with noise sensitivity were older compared with control dogs. Even though dog personality has been observed to be rather stable from one year onwards [50], our observation suggests that fearfulness may be reduced along with age, possible due to habituation and life experiences. Dogs may develop a coping strategy for frightening situations, partially with the help of the owner. Owners may also learn that their dogs do not like new people or new situations and may start avoiding these situations. This however does not seem to be the case with noise sensitivity, as noise-sensitive dogs were older compared with dogs with no fear of loud noises. Noise sensitive dogs have been found to be older compared with control dogs in other studies as well [8]. In addition, one study found that dogs over 10-years-old have more severe fear reactions compared with younger dogs [51]. The median onset age for noise sensitivity in our study was 2 years, which partially explains the association. Fear of loud noises seems to differ from, for example, a fear of strangers, in the sense that habituation to the loud noises does not seem to happen; instead, fear of noises might even get more severe during aging [51].

The presence of other dogs may work as stress resilience. We found that fearful dogs came from single-dog houses more often than non-fearful dogs. An earlier study reported that dogs from multi-dog homes have less pronounced reactivity and more rapid recovery of the dog’s HPA response compared with dogs from single-dog houses after exposure to a simulated thunderstorm [52]. The difference was only observed in the cortisol recovery, and the behavioral responses of dogs from multi- and single-dog houses did not differ [52]. We have earlier found that dogs with stereotypic tail-chasing have fewer dog companions at home compared with dogs with no tail chasing [35]. Although we speculated earlier that this difference might be an artifact (of owners from multi-dog houses being able to pay less attention to each dog compared with single-dog homes), the fact that this difference is again observed in relation to anxiety-related behavior suggests that living with other dogs may work as a protective factor against several stressors. Social support has long been known to be a buffering factor against stressful events in humans [53], whereas social isolation in monogamous prairie voles is a stress-inducing factor [54,55].

Owners' first dogs were more likely to develop noise sensitivity compared with owners' later dogs, which may reflect the owner experience in training, desensitizing, or more careful selection of later puppy personality or breed. Owners' first dogs have also been found to have a higher incidence of noise sensitivity [25,26]. We also found that dogs living with several adults were more fearful than those living in families with only one adult. The possible explanation for this association is unclear.

### Sex, sterilization status, and other factors

Females and males differed in fearfulness, but not in noise sensitivity. Females were more often found to be fearful compared with males. Clear sex difference has been found earlier, especially both for fearfulness and phobias, where females were diagnosed with having general fearfulness and phobias more often [56]. Correspondingly, males have been reported to be bolder in earlier studies [57,58]. In our study, noise sensitive dogs were observed to be sterilized/neutered more often compared with control dogs. This increase in anxiety due to hormonal level change has been observed in earlier studies as well [8], and early neutering has been shown to predispose to several anxieties [59]. We also observed a significant interaction term between sex and sterilization. Intact females had higher probability to fear loud noises compared with intact males. In other words, although sterilization seems to be a risk factor for noise sensitivity, it is a similar-sized risk factor for both sexes. Dogs living mostly indoors were more fearful compared with dogs living mostly in kennels/outdoors in our study. An earlier study found noise-phobic dogs to live indoors more often compared with control dogs [27]. This may be a real observation, or alternatively, dogs living mostly outdoors are not as often observed by the owners, generating a reporting bias.

Dog breeding includes practices, where breeders use several co-owned females in breeding that live somewhere other than at breeders’ homes permanently. However, these females are usually taken into breeders' homes for delivery, where the bitches take care of puppies until those are 7–8 weeks old on average. This change of environment just before the birth may cause stress for these females, and we wanted to investigate if the birthplace has any effect on a puppy’s future fearfulness. We observed a significant effect of the birthplace on offspring behavior. However, the direction was the opposite than expected; the offspring born from females that were brought to breeders’ homes for delivery were *less* fearful compared to offspring whose mothers gave birth at permanent homes. The same phenomenon was observed in noise sensitivity. One of the potential explanations could be the difference in maternal care between the environments, and we actually did find differences in maternal care between delivery environments. However, females who delivered at their permanent homes took better care of their offspring than mothers that were taken into breeders' homes, which does not explain why permanent home-delivered dogs are more fearful. Another explanation for the observation may be the beneficial effects of mild stress. The change of environment for the mother just before delivery is a potential stressor, and this stress may mediate via the mother’s behavior to the offspring. Several animal studies show beneficial effects of early exposure to mild stress, such as decreased anxiety in adult offspring [60,61].

Dogs with noise sensitivity and anxiety comorbidity spend less time alone on average compared to dogs with no noise sensitivity. Dogs with noise sensitivity may also have separation anxiety symptoms, as it has been found that these anxieties often co-occur [62,63]. Due to these symptoms, owners may leave these dogs alone for shorter periods. Owners of solely fearful dogs engaged less in activities such as training, competing, hunting, etc., compared to non-fearful dogs. The dogs' fearfulness may actually restrict these activities, and owners may not be very motivated to compete with fearful dogs, as it has been shown that bold dogs are more successful in working dog competitions [64].

## Conclusions

This questionnaire study revealed several environmental factors that are related to canine anxiety. Many of these factors have also been found to be important in the personality development and anxiety resilience in other mammals, including humans. Both of the largest environmental factors associating with fearfulness were related to early life. The amount of socialization has long been known to affect fearfulness in dogs. However, our observation of the role of maternal care is novel and warrants mode detailed studies in future. Maternal care in dogs in this study was estimated using only one question, and more detailed research on the variation within and between breeds and on the important components of maternal care using the observational approach is needed. The estimation of maternal traits is used actively in several production animal breeding-programs, and maternal qualities should be estimated and used in future dog breeding programs. The major factor associating with noise sensitivity and separation anxiety was the amount of daily exercise. Although some indications of this association can be found from earlier literature, this is the first time when both the amount and the type of exercise can be identified as both having a significant association with anxiety in dogs. A replication study in a larger sample or by linking the serotonin pathway and exercise to anxiety via a functional approach is needed. If further studies can show a causal connection, then exercise could potentially provide a feasible means to improve the welfare of anxious dogs.

## Supporting Information

S1 Appendix The questionnaire used to collect the data. (DOCX)
