# Supplementary material for: Early Life Experiences and Exercise Associate with Canine Anxieties
Source: PLoS One. 2015 Nov 3;10(11):e0141907. doi: 10.1371/journal.pone.0141907 (PMC4631323; doi:10.1371/journal.pone.0141907)
Supplement: S1 Appendix — (DOCX) [file pone.0141907.s001.docx]

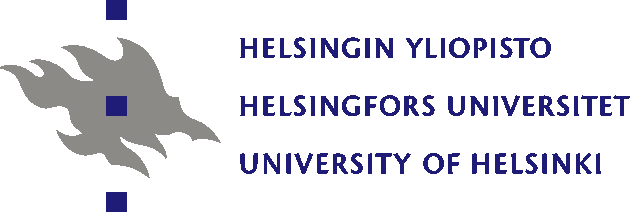
**Genetics behind behaviour**

| Name of the owner: |
| --- |
| Address: |
| Phone number: |
| Email address: |
| Breed: |
| Dog’s name (official and call name): |
| Registration number: |
| sex:  male  female |
| Is the dog spayed/neutered:  yes   no |
| If you answered yes, at what age is your dog spayed/neutered: |
| Do you have a blood sample from your dog in Hannes Lohi's research group's DNA bank?:  yes  no  I don’t remember |

This questionnaire includes questions which deal with fear, shyness, aggressiveness, noise phobia and separation anxiety. Generalized fear and noise phobia are known to have a heritable component, and the aim of this study is to find the genetic factors behind these behavioural traits in dogs. In addition to filling this questionnaire, a blood sample (or cheek swap) from your dog is needed. This study is conducted at the Helsinki University and at The Folkhälsan Institute of Genetics, Finland.

Please answer all the questions; even though your dog may not express shyness, aggressiveness or noise phobia, as we also need ‘control’ dogs in the study. If you are submitting forms for multiple dogs, you need to complete one questionnaire for each dog. All information submitted is strictly confidential. Neither you nor your dogs will be identified at any time.

You can find more information on the question by taking your pointer above the red question mark.

This questionnaire is a modified version from K9BEHAVIOURAL GENETICS QUESTIONNAIRES (Univ. of California, San Francisco & University of Pennsylvania, Philadelphia).

If you have any questions, please contact [katriina.tiira@helsinki.fi](mailto:katriina.tiira@helsinki.fi) , or hannes.lohi@helsinki.fi

**Background information**

Fear and shyness is affected not only genes but also by dog’s experiences, particularly in the puppyhood. Please take time in answering the following questions from your dog’s history. You may have to contact to the breeder of your dog for answering some questions. You can find more information on the question by taking your pointer above the red question mark.

1. At what age did the dog enter to your household?

2. Does the dog have any previous owners? Why didn’t the previous owners keep the dog?

3. Dog was acquired from  home breeder

home breeder (showline breeding)

home breeder (working line breeding)

Large kennel

Larger kennel (show dogs)

Larger kennel ((working dogs)

Other

4. Has the mother of your dog given birth to the litter at her home, or somewhere else (in the breeder’s kennel)

in her home

in the breeder’s kennel, or somewhere else (=not at the dog’s home)

I don’t know

5. The birth of your dog. When your dog was born, were there any problems in the labour?

Yes, there were problems, please spesify

no

I don’t know

6. Separation from the mother - at what age? At what age was your dog separated from its mother? Usually this happens around 4-9 weeks, however it is not always the same as the age when puppy enters new home.

under 4 weeks

at the age of 4 weeks

at the age of 5 weeks

at the age of 6 weeks

at the age of 7 weeks

at the age of 8 weeks

at the age of 9 weeks

at the age of 10-12 weeks

over 12 weeks or older

is still living in the same household with its mother

7. How did the mother of your dog take care of the puppies?

I don't know

Mother took extremely good care of the puppies and spend a lot of time with the puppies

Mother took good care of the puppies

Mother took relatively good care of the puppies, but sometimes it had to pushed back to spend time with the puppies

At the beginning the mother spent some time with its puppies, but later started to avoid being with puppies

Mother did not want to spent time with the puppies, even at the beginning, but nursed enough so the puppies were not taken from its mother

Mother did not take care of puppies; puppies were taken to a surrogate mother, or they were bottle fed

How did the mother of your dog take care of the puppies? If any of the obtions was not suitable, please tell in your own words

8. The socialization period: has the dog experienced the following events during the period between 7 weeks-3 months? How often?

*Events How often?*

Met strange adult dogs

very often (several times per day)

often (twice a week-once a day)

sometimes (twice a month-twice a week)

seldom (1-2 times at puppyhood - twice a month)

rarely (less than 1-2 times during puppyhood)

never

Met strange women

very often (several times per day)

often (twice a week-once a day)

sometimes (twice a month-twice a week)

seldom (1-2 times at puppyhood - twice a month)

rarely (less than 1-2 times during puppyhood)

never

Met strange men

very often (several times per day)

often (twice a week-once a day)

sometimes (twice a month-twice a week)

seldom (1-2 times at puppyhood - twice a month)

rarely (less than 1-2 times during puppyhood)

never

Met strange children

very often (several times per day)

often (twice a week-once a day)

sometimes (twice a month-twice a week)

seldom (1-2 times at puppyhood - twice a month)

rarely (less than 1-2 times during puppyhood)

never

Visited city (or other place with traffic &

many people)

very often (several times per day)

often (twice a week-once a day)

sometimes (twice a month-twice a week)

seldom (1-2 times at puppyhood - twice a month)

rarely (less than 1-2 times during puppyhood)

never

Travelled by car

very often (several times per day)

often (twice a week-once a day)

sometimes (twice a month-twice a week)

seldom (1-2 times at puppyhood - twice a month)

rarely (less than 1-2 times during puppyhood)

never

Travelled by buss

very often (several times per day)

often (twice a week-once a day)

sometimes (twice a month-twice a week)

seldom (1-2 times at puppyhood - twice a month)

rarely (less than 1-2 times during puppyhood)

never

9. Your household includes       adults and       children

10. How many dogs do you have at the moment (please mention breed, sex and age of the dogs)?

11. Is the dog in question your first? Second? 10th?

12. Dog lives  indoors  outside/ in the kennel

partly inside/partly in the kennel/outside

other

13a. Do you engage any activities with your dog? Please specify?

13b. Activities with the dog – how much do you spend time in activities mentioned above? (daily walking excluded)

zero

once a year

1-2 times / half a year

1-2 times / month

1-2 times / week

2-4 times / week

nearly daily

14. What does your dog eat?

home food (specially made for the dog)

home food (leftovers)

commercial dog food (pet shop)

commercial dog food (supermarket)

something else, please specify

15. Do you give your dogs extra vitamins etc.?

Regularly

Sometimes

Never

16. How many times does your dog get exercise in a typical day?

three times or more

twice a day

once a day

dog is outside all the time

something else, please specify

During the daily walks, is your dog

on the leach during the whole walk

dog is leached part of the walk, and partly dog is allowed to run free

dog is mostly allowed to run free during the walks

17. How many hours/minutes does your dog get exercise in a typical day?

three hours or more

2-3 hours

1-2 hours

30 min-1 hour

less than 30 min

18. How much does your dog spend alone in the house/kennel during the average working day?

0 hours

0-1 hours

1-3 hours

3-6 hours

6-8 hours

8-9 hours

9-10 hours

10 hours or more

**Shyness/fearfulness**

The following questions deal with dog’s potential fearful reactions towards strangers, unfamiliar dogs and also dog’s behaviour in new/strange places or situations.

If your dog’s behaviour is changed at some point, please describe that change in the section ‘Changed behaviour’.

Think how your dog usually reacts when meeting a stranger. If your dog shows shyness towards strangers, please mark **YES, my dog shows fearful behaviour**, **and then mark those appropriate behaviour(s) which best describe the behaviour of your dog.** If none of the behaviours listed below describe well your dog’s behaviour, you can add a suitable behaviour to the list**. If your dog does not behave fearfully, mark NO, and mark how your dog does behave when meeting a stranger.**

**19. Stranger**

**YES, DOG REACTS FEARFULLY**  **NO, DOG DOES NOT REACT FEARFULLY**

**When the dog meets a strange person, it…**

withdraws

barks (is not going towards person)

growls (is not going towards person

tail low / between the legs

not willing to make contact

stays close to the owner

barks / growls AND goes towards a stranger

**How often does the dog react fearfully?**

Always, 100% of the times

Almost always, 60-100% of the times

Often, 40-60% of the times

Rarely, 0-40% of the times

If you answered NO, please specify how your dog behaves when meeting a stranger

if allowed, always goes to greet the person

jumps, licks, is very excited

takes relaxed contact, sniffs and tail is wagging

sniffs, bot loses its interest soon

is not interested on people, but does not back up or mind if a person wants to pet the dog

is not interested

barks, growls

**something else**

If the behaviour has changed at some point, please describe how your dog used to behave and how does it behaves now?

**Changed behaviour**

Former behaviour

Now

**20. Unfamiliar dog**

Think how your dog usually reacts when meeting unfamiliar dogs. If your dog is shy when meeting unfamiliar dogs, please mark **YES, my dog shows fearful behaviour**, **and then mark those appropriate behaviour(s) which best describe the behaviour of your dog.** If none of the behaviours listed below describe well your dog’s behaviour, you can add a suitable behaviour to the list**. If your dog does not behave fearfully, mark NO, and mark how your dog does behaves.**

**Does your dog show shyness or fear when meeting an unfamiliar dog?**

**YES, DOG REACTS FEARFULLY  NO, DOG DOES NOT REACT FEARFULLY**

**When the dog meets an unfamiliar dog, it…**

withdraws

barks (is not going towards the dog)

growls (is not going towards the dog)

tail low / between the legs

not willing to make contact

stays close to the owner

barks / growls AND goes towards a stranger

**If you answered YES, please specify how does your dog react?**

**How often does the dog react fearfully?**

Always, 100% of the times

Almost always, 60-100% of the times

Often, 40-60% of the times

Rarely, 0-40% of the times

If you answered NO, please specify how your dog behaves when meeting an unfamiliar dog

eager to meet, always friendly

eager to meet, but loses its interest soon

usually friendly, but responds aggressively if another dog shows aggressive behaviour

usually friendly, but sometimes starts a fight

usually growls

usually barks

barks / growls AND goes towards a dog

indifferent, not interested in other dogs

something else, please specify

**Changed behaviour**

Former behaviour

Now

**21. New situation, new environment**

Think how your dog usually reacts in new situations or places. If your dog shows fear is stressed (panting, shaking, intense peeling) please mark **YES,**  **and then mark those appropriate behaviour(s) which best describe the behaviour of your dog.** If none of the behaviours listed below describe well your dog’s behaviour, you can add a suitable behaviour to the list**. If your dog does not behave fearfully, mark NO, and mark how your dog does behaves.**

**In the case your dog shows fear only at the veterinary clinic, please do not take that into account.**

**Does your dog show fear or stress in a new situation or in a new environment?**

**YES, DOG REACTS FEARFULLY  NO, DOG DOES NOT REACT FEARFULLY**

**In a new situation /in a new environment my dog…**

wants out of the situation / new space

barks

tail low

stays still, does not want to explore the new environment

stays close the owner (not under any command)

walks low

pants

trembls

**How often does the dog react fearfully?**

Always, 100% of the times

Almost always, 60-100% of the times

Often, 40-60% of the times

Rarely, 0-40% of the times

**Changed behaviour**

Former behaviour

Now

If you answered NO, please specify how your dog behaves in a new situation or environment

tail in high position

tail below the backline, but not between the legs

dog is curious, eager to inspect

is able to eat and sleep at the new place

is calm, rather indifferent

something else, please specify

**Other things that might be related to my dogs behaviour towards strange persons, dogs, or behaviour in new situations:**       **Aggressiveness**

The following questions deal with the dog’s aggressive behaviour towards strangers, dogs and owner / family member.

If the dog does not show aggressive behaviour, mark NO. If the dog shows aggressive behaviour, please mark the frequency of that particular behaviour on the scale 1-4. For example, if the dog almost always barks other dogs, then mark number 2 on ‘bark’ in question 16.

4= Always (100% of the occasions)

3= Nearly always (60-100% of the occasions)

2= Often (40-60% of the occasions)

1= Seldom (1-40% of the occasions)

0=never

**Aggressive behaviour**

| target | **NO reaction** | Growl | Bark  (aggr.) | Snap  (no skin contact) | Bite  (Connects with skin) |
| --- | --- | --- | --- | --- | --- |
| 22. Strange person |  |  |  |  |  |
| 23. Familiar person |  |  |  |  |  |
| 24. Owner/ member of the family |  |  |  |  |  |
| 25. Strange dog |  |  |  |  |  |
| 26. Familiar dog |  |  |  |  |  |

**Changed behaviour:**

Former behaviour:

Now :

Are there any other issues that might be related to the aggressive behaviour of your dog which might be relevant concerning this study?      **Noise phobia**

The following questions deal with your dog’s reaction to loud noises. If your dog **does not react** on particular noise in question, mark **‘No’**. If your dog’s behavior has changed at any time, please, describe the former and present behavior under ‘Changed behavior’.

**27. THUNDERSTORM**

**YES  NO REACTION  DON’T KNOW**

**If you answered YES, please mark one or more behaviours that describe your dog's reaction**

salivate

defecate

urinate

destroy

escape

pant

hide

tremble

vocalize

pace

freeze

tail low/ between legs

**How often does the dog react to thunderstorm?**

Always, 100% of the times

Almost always, 60-100% of the times

Often, 40-60% of the times

Rarely, 0-40% of the times

**Changed behaviour**

Former behaviour

Now

**28. FIREWORKS**

**Does your dog react to fireworks?**

**YES  NO REACTION  DON’T KNOW**

**If you answered YES, please mark one or more behaviours that describe your dog's reaction**

salivate

defecate

urinate

destroy

escape

pant

hide

tremble

vocalize

pace

freeze

tail low/ between legs

**How often does the dog react to fireworks?**

Always, 100% of the times

Almost always, 60-100% of the times

Often, 40-60% of the times

Rarely, 0-40% of the times

**Changed behaviour**

Former behaviour

Now

**29. GUNSHOTS**

**YES  NO REACTION  DON’T KNOW**

**If you answered YES, please mark one or more behaviours that describe your dog's reaction**

salivate

defecate

urinate

destroy

escape

pant

hide

tremble

vocalize

pace

freeze

tail low/ between legs

**How often does the dog react to gunshot?**

Always, 100% of the times

Almost always, 60-100% of the times

Often, 40-60% of the times

Rarely, 0-40% of the times

**Changed behaviour**

Former behaviour

Now

**30. OTHER NOISES (vacuum cleaners, leaf blowers, sirens, alarm systems etc.)**

**YES  NO REACTION  DON’T KNOW**

**If you answered YES, please mark one or more behaviours that describe your dog's reaction**

salivate

defecate

urinate

destroy

escape

pant

hide

tremble

vocalize

pace

freeze

tail low/ between legs

**How often does the dog react to gunshot?**

Always, 100% of the times

Almost always, 60-100% of the times

Often, 40-60% of the times

Rarely, 0-40% of the times

**Changed behaviour**

Former behaviour

Now

**31**. Is the intensity of the dog’s reaction different when the dog is engaged in activities such as agility, obedience training, herding livestock, retrieving fowl?

a.  Yes, it is non-existent

b.  Yes, it is less severe

c.  No, it is the same

d.  Yes, it is more severe

**32**. How frequently do noise events such as thunder, fireworks or gunshots occur in the dog’s environment?

a.  Never

b.  Infrequently (few times a year)

c.  Regularly (averaging once a month or so)

d.  Frequently (a few times a month or more)

If you answered **b, c or d, to 31**what are the noises?

**33**. Has the dog ever been treated for noise sensitiveness or phobias? Please check all relevant choices below?

Acerpromazine

Rescue Remedy

Benzodiazepine, (Valiumilla or Xanaxilla

Other ’natural’ or ‘holistic’ remedies

Desensitization (tapes, CDs, videos)

Other?:

**34**. Do you have additional comments about your dog’s reaction to noises, or is there anything else about his or her behaviour when exposed to noise that you think we should know:

**Separation anxiety**

**35**. Does your dog exhibit separation anxiety when left alone?

**YES**  **/ NO**

If you answered yes, please explain how the dog behaves:
